# Association Between *AGT* M235T and Left Ventricular Mass in Vietnamese Patients Diagnosed With Essential Hypertension

**DOI:** 10.3389/fcvm.2021.608948

**Published:** 2021-02-19

**Authors:** Tuan Thanh Tran, Thao Phuong Mai, Ha Chau Bich Tran, Linh Hoang Gia Le, Hoang Anh Vu, Trang Kim Tran, Sy Van Hoang, Hoa Ngoc Chau, Minh Duc Do

**Affiliations:** ^1^Department of Internal Medicine, Faculty of Medicine, University of Medicine and Pharmacy at Ho Chi Minh City, Ho Chi Minh City, Vietnam; ^2^Department of Physiology-Pathophysiology-Immunology, Faculty of Medicine, University of Medicine and Pharmacy at Ho Chi Minh City, Ho Chi Minh City, Vietnam; ^3^Center for Cardiovascular Medicine, University Medical Center, Ho Chi Minh City, Vietnam; ^4^Center for Molecular Biomedicine, University of Medicine and Pharmacy at Ho Chi Minh City, Ho Chi Minh City, Vietnam

**Keywords:** essential hypertension, echocardiography, left ventricular mass, Vietnam, *AGT* M235T

## Abstract

**Background:** Increasing left ventricular mass in hypertensive patients is an independent prognostic marker for adverse cardiovascular outcomes. Genetic factors have been shown to critically affect left ventricular mass. *AGT* M235T is one of the genetic polymorphisms that may influence left ventricular mass due to its pivotal role in the regulation of plasma angiotensinogen level as well as hypertension pathophysiology in Asian populations. Currently, how M235T affects left ventricular mass is not well-described in Vietnamese hypertensive patients. This study aimed to investigate the association between M235T and left ventricular mass in Vietnamese patients diagnosed with essential hypertension.

**Materials and Methods:** *AGT* M235T genotyping and 2D echocardiography were performed on 187 Vietnamese subjects with essential hypertension. All the ultrasound parameters were obtained to calculate the left ventricular mass index according to the American Society of Echocardiography and the European Association of Cardiovascular Imaging 2015 guidelines. Other clinical characteristics were also recorded, including age, gender, duration of hypertension, hypertensive treatment, lifestyle, renal function, fasting plasma glucose, and lipid profile.

**Results:** MT and TT genotypes were determined in 30 and 157 subjects, respectively. *AGT* M235T genotype, duration of hypertension, body mass index, and ejection fraction statistically affected the left ventricular mass index, which was significantly greater in TT compared to MT carriers after adjusting for confounding factors.

**Conclusion:** The TT genotype of *AGT* M23T was associated with greater left ventricular mass in Vietnamese patients diagnosed with essential hypertension.

## Introduction

Essential hypertension is a major health-care burden globally, despite the availability of efficient antihypertensive agents (1). Increasing left ventricular mass (LVM) identified by echocardiography in hypertensive patients has been well-proven to be a strong independent predictor of adverse cardiovascular outcomes, including sudden cardiac death, coronary artery diseases, stroke, and heart failure (2). Besides the severity and duration of hypertension, plenty of other factors have been found to affect LVM, including age, gender, ethnicity, and genetic elements, as well as comorbidities such as obesity, diabetes, and chronic kidney disease (3, 4).

Genetic components have been shown to be associated with LVM in hypertensive patients (5–8). Variants of renin-angiotensin-aldosterone (RAA) genes have attracted an enormous amount of attention due to the important physiological roles of the RAA system (9). The RAA hormonal system regulates blood pressure and fluid and electrolyte balance, together with vascular resistance (10). Angiotensinogen (AGT) is first fragmented by renin to produce angiotensin I, the angiotensin-converting enzyme (ACE) then converts angiotensin I to the active form angiotensin II. Angiotensin II binds to receptors leading to the synthesis of aldosterone, which promotes the reabsorption of water and salt in the renal tubes (11). The RAA system affects LVM not only indirectly through blood pressure but also directly by the action of angiotensin II on cardiomyocytes (12). Numerous studies have been conducted to elucidate the association between RAA genetic polymorphisms and LVM (13–15). Among candidate variants, *AGT* M235T is one of the more promising markers showing an association with LVM in hypertensive patients. *AGT* M235T is characterized by the substitution of Threonine for Methionine in codon 235, and is well-proven to affect the plasma angiotensinogen concentration (16). Interestingly, the T allele of M235T, which is related to higher plasma AGT, is well-associated with hypertension in Africans and Asians, such as Nigerian, Egyptian, Malaysian, and Japanese populations (17–21), but this association remains controversial in Caucasians (22–24). Similarly, the unfavorable effects of the T allele in increasing LVM have been observed in Chinese but not Russians or Americans (25–27). Mechanisms that underlie how *AGT* M235T affects LVM are still elusive. One possibility is the greater plasma AGT level in TT-carriers resulting in an excessive amount of angiotensin II, which regulates the growth of cardiomyocytes independently of blood pressure (28). Another possibility is the linkage disequilibrium between M235T and other driving variants leading to the increase in LVM.

So far, the association between RAA genetic variants and LVM in Vietnamese hypertensive patients has not been elucidated. This study aimed to identify the association between *AGT* M235T and LVM in Vietnamese patients diagnosed with essential hypertension.

## Materials and Methods

### Subjects

This study was approved by the Ethical Committee of the University of Medicine and Pharmacy at Ho Chi Minh City, Vietnam. Patients, either newly diagnosed or previously diagnosed with essential hypertension and on antihypertensive agents were recruited to this study, which took place in the University Medical Center between January 2019 and May 2019. All patients were self-identified as Kinh Vietnamese and were randomly selected with k = 10 from the daily registered list for cardiovascular examination. Patients were excluded from the study if they had been diagnosed with diabetes or had a history of angina or myocardial infarction, or their estimated glomerular filtration rate was < 60 ml/min/1.73 m^2^ of the body surface. The patients were counseled, and they agreed to participate in the study by providing written informed consent. The age, gender, height, and body weight of the patients were recorded. Body weight was measured using digital scales (HN-289; Omron, Tokyo, Japan) in clothing without shoes. Height was determined without shoes on a wall-mounted stadiometer (HM200PW; Charder Medical, Taichung, Taiwan) with mandible plane parallel to the floor. Body mass index (BMI) was calculated as weight (kg) divided by the square of height (m) (29); body surface area was estimated by the square root of the height (cm) multiplied by the weight (kg) divided by 3,600 (30). All information including age at hypertension diagnosis, duration of hypertension, antihypertensive medication, high-salt diet, smoking, alcohol use, exercise, family history of hypertension, and later, blood pressure and echocardiography parameters, were documented. A high-salt diet was assessed using the questionnaire developed by the Salt Consumption Survey in the Republic of Moldova Study Group (31). Alcohol use and smoking were assessed using the WHO STEPS Instrument for non-communicable diseases Risk Factor Surveillance (32). Blood pressure was measured automatically by a digital sphygmomanometer (JPN600, Omron, Tokyo, Japan). Newly diagnosed hypertensive patients were identified based on the diagnostic criteria of the European Society of Cardiology and the European Society of Hypertension 2018 guidelines for the management of arterial hypertension (33). For hypertensive patients who were equal to or < 35 years old, renal artery ultrasound, abdominal ultrasound, blood electrolyte, thyroid-stimulating hormone, plasma renin and aldosterone, 24-h urinary catecholamine, and cortisol tests were performed to investigate the hypertensive etiology. If any result of these tests was abnormal, the participant was excluded. Hypertensive patients who were > 35 years old were excluded from the study if they were suspected of having secondary hypertension, based on their history and a physical examination. For newly diagnosed patients, a cardiac ultrasound was performed only when the hypertensive diagnosis was determined in the second visit. Following the cardiac ultrasound, patients with moderate to severe valvular heart disease, moderate to severe pulmonary hypertension, hypertrophic cardiomyopathy, myocardial ischemia [defined as abnormalities in wall motion (hypokinesia/akinesia)], aneurysm, or ejection fraction (EF) < 55% were also excluded from the study. Finally, 2 mL of venous blood was drawn from 187 of the participants for genetic analysis. Other laboratory measurements were obtained, such as fasting plasma glucose (mg/dl), estimated glomerular filtration rate (GFR) from serum creatinine, total cholesterol (mmol/L), HDL-cholesterol (mmol/L), LDL-cholesterol (mmol/L), and triglyceride (mmol/L).

### Echocardiography

Two-dimensionally guided M-mode echocardiography was performed using the Affiniti 50G ultrasound system (Philips, Amsterdam, The Netherlands) with a 2–4 MHz transducer at the Cardiology Imaging Unit of the University Medical Center at Ho Chi Minh City. All the echocardiography parameters used for further analysis were the average of two measurements obtained blindly by two independent cardiologists with proper echocardiographical expertise according to the recommendations of the American Society of Echocardiography and the European Association of Cardiovascular Imaging 2015 guidelines (34, 35). Left ventricular parameters, such as left ventricular internal dimension at end-diastole (LVIDd) and in systole (LVIDs), posterior wall thickness at end-diastole (LPWd) and in systole (LPWs), and interventricular septal thickness at end-diastole (IVSd) and in systole (IVSs) were measured for three beats, and average values were collected for further calculations. LVM was calculated from the measurements of the left ventricle (LV) using the equation: LVM = ((IVSd+LVDd+LPWd)^3^-(LVDd)^3^) × 1.04 × 0.8 + 0.6 (g). LVMI (left ventricular mass index) was identified by the formula: LVMI = LVM/BSA (g/m^2^). Left ventricular hypertrophy was defined as LVMI >115 g/m^2^ for men or 95 g/m^2^ in women (34). Relative wall thickness (RWT) was calculated with the formula RWT = 2xLPWd/LVDd. RWT was considered abnormal if it was > 0.42. Four left ventricular geometric patterns were identified based on RWT and LVMI: normal geometry, concentric remodeling, eccentric hypertrophy, and concentric hypertrophy. LV geometry was defined as concentric hypertrophy (elevated LVMI and RWT), concentric remodeling (normal LVMI and elevated RWT), eccentric hypertrophy (increased LVMI and normal RWT), and normal geometry (normal LVMI and RWT). EF was assessed using the Teichholz method (36). Supplementary Figure 1 represents how echocardiography measurements were documented.

### Genetic Analysis

Genomic DNA was extracted from patients' leukocytes using a GeneJet^™^ Whole Blood Genomic DNA Purification Mini Kit (Thermo Fisher Scientific, Waltham, MA, USA). Four primers were designed for a tetra-primer allele-specific polymerase chain reaction (PCR) to identify M235T based on the sequence of the *AGT* gene (Genebank NG_008836). The length of control bands was 282 bp. Allele T and allele M were determined by the appearance of 118 bp and 204 bp bands, respectively. The sequences of primers are listed in Supplementary Table 1.

PCRs were performed using a Mastercycler@proS (Eppendorf, Hamburg, Germany) with the following conditions: one cycle at 98°C for 3 min and 40 cycles of denaturation at 98°C for 20 s, annealing at 56°C for 20 s, extension at 72°C for 30 s, and final extension at 72°C for 2 min. The product length of PCR was identified by electrophoresis on 2% agarose gels with Diamond^™^ Nucleic Acid Dye (Promega, Madison, WI, USA).

### Data Analysis

The data were encrypted to ensure blinded analysis. Data were input using Epidata 3.1 and subsequently analyzed using Stata 13.3 software. *T-*test and Chi-squared tests were performed for statistical analysis. Univariate analysis and multivariate logistic regression were used to assess the association between patients' characteristics and LVMI. All tests were considered significant if *p* ≤ 0.05.

## Results

TT, MT, and MM genotypes of *AGT* M235T were found in 157, 30, and 0 individuals, respectively. The percentage of T and M alleles were 92 and 8%. The distribution of *AGT* M235T was under Hardy Weinberg equilibrium, with *p* = 0.49. Patients' characteristics are described in Table 1. The mean age of the participants was 46, with an average duration of essential hypertension of 3.5 years. Males comprised 55% of the population studied. All the clinical and laboratory characteristics were not significantly different between MT and TT-genotyped participants, except for triglyceride concentration and alcohol usage. Factors that were known to influence LVM, including age, gender, treatment with ACE inhibitors or angiotensin II receptor blockers, BMI, and eGFR, were not statistically different between the two genotypes.

**Table 1:** Characteristics of hypertensive patients by M235T genotypes.

**Characteristics**	**Population** **(*n =* 187)**	**MT** **(*n =* 30)**	**TT** **(*n =* 157)**	***P-*value**
Age (years) (Mean ± SD)	46.0 ± 9.5	44.0 ± 9.9	46.4 ± 9.4	0.25
Male gender (%)	55.1%	43.3%	57.3%	0.16
Age at diagnosis (years) (Mean ± SD)	42.5 ± 9.8	40.6 ±11.4	42.9 ± 9.4	0.24
Duration of hypertension (years) (Mean ± SD)	3.5 ± 4.3	3.6 ± 3.6	3.5 ± 4.4	0.91
Use of antihypertensive medication (%)	83.4%	86.7%	82.8%	0.60
ACE inhibitor (%)	17.7%	16.0%	18.1%	1.00
Angiotensin II receptor blockers (%)	60.0%	72.0%	57.1%	0.17
Calcium channel blockers (%)	57.7%	44.0%	61.0%	0.12
Diuretics (%)	15.4%	12.0%	16.2%	0.76
Beta-blockers (%)	22.3%	36.0%	19.1%	0.07
BMI (kg/m^2^) (Mean ± SD)	24.4 ± 2.8	23.9 ± 3.6	24.5 ± 2.6	0.39
BSA (m^2^) (Mean ± SD)	1.7 ± 0.2	1.7 ± 0.2	1.7 ± 0.2	0.26
High salt intake (%)	64.7%	50.0%	67.5%	0.07
Smoking (%)	15.0%	13.3%	15.3%	1.00
Alcohol usage (%)	48.1%	26.7%	52.2%	0.01^*^
Sedentary lifestyle (%)	65.8%	76.7%	63.7%	0.17
Family history of hypertension (%)	71.7%	70.0%	72.0%	0.83
Fasting plasma glucose (mg/dl) (Mean ± SD)	98.2 ± 10.6	98.4 ± 10.6	98.2 ± 10.6	0.94
eGFR (ml/min/1.73 m^2^) (Mean ± SD)	83.8 ± 13.0	85.3 ± 17.8	83.5 ± 12.0	0.60
Total cholesterol (mmol/L) (Mean ± SD)	5.5 ± 1.3	5.3 ± 1.2	5.5 ± 1.3	0.39
HDL cholesterol (mmol/L) (Mean ± SD)	1.2 ± 0.3	1.2 ± 0.3	1.2 ± 0.3	0.88
LDL cholesterol (mmol/L) (Mean ± SD)	3.6 ± 0.9	3.5 ± 0.9	3.6 ± 0.9	0.67
Triglyceride (mmol/L) (Mean ± SD)	2.5 ± 1.8	1.9 ± 0.8	2.6 ± 1.9	0.00^*^

*BMI, body mass index; BSA, body surface area; eGFR, estimated glomerular filtration rate*.

* *statistically significant*.

Echocardiography parameters are listed in Table 2. All the measures were not statistically different between TT and MT genotypes, except for LVMI, which was significantly greater in TT compared to MT-carrying subjects. The distribution of left ventricular geometric patterns is listed in Table 3. No significant differences were found in left ventricular geometric patterns between the genotypes. The majority of LV geometric patterns were normal in both MT and TT groups.

**Table 2:** M-mode echocardiographic measures in hypertensive patients by M235T genotypes.

**Measures**	**Population**	**MT**	**TT**	***P-*value**
**(Mean ± SD)**	**(*n =* 187)**	**(*n =* 30)**	**(*n =* 157)**	
IVSd (mm/m^2^)	5.5 ± 1.0	5.3 ± 1.2	5.6 ± 1.0	0.13
IVSs (mm/m^2^)	7.7 ± 1.3	7.3 ± 1.5	7.8 ± 1.2	0.08
LVIDd (mm/m^2^)	28.1 ± 3.0	28.4 ± 3.1	28.0 ± 3.0	0.58
LVIDs (mm/m^2^)	17.3 ± 2.3	17.4 ± 1.9	17.2 ± 2.3	0.68
LPWd (mm/m^2^)	5.5 ± 1.0	5.3 ± 1.1	5.6 ± 1.0	0.22
LPWs (mm/m^2^)	8.5 ± 1.5	8.5 ± 1.4	8.5 ± 1.5	0.77
EF (%)	68.7 ± 5.8	68.2 ± 6.4	68.8 ± 5.7	0.61
LVMI (g/m^2^)	89.7 ± 20.9	82.4 ± 20.4	91.1 ± 20.7	0.04^*^

*EF, ejection fraction; IVSd, interventricular septal thickness at end-diastole; IVSs, interventricular septal thickness in systole; LVIDd, left ventricular internal dimension at end-diastole; LVIDs, left ventricular internal dimension in systole; LPWd, posterior wall thickness at end-diastole; LPWs, posterior wall thickness in systole; LVMI, left ventricular index*.

* *statistically significant*.

**Table 3:** Left ventricular geography in hypertensive patients with AGT genotypes.

**Geometric pattern**	**Population**	**MT**	**TT**	***P-*value**
	**(*n =* 187)**	**(*n =* 30)**	**(*n =* 157)**	
Normal	49% (*n =* 92)	57% (*n =* 17)	47% (*n =* 74)	0.82
Concentric remodeling	28% (*n =* 52)	27% (*n =* 8)	28% (*n =* 44)	
Concentric hypertrophy	11% (*n =* 21)	6% (*n =* 2)	12% (*n =* 19)	
Eccentric hypertrophy	12% (*n =* 22)	10% (*n =* 3)	13% (*n =* 20)	

Univariate analysis showed that the duration of hypertension, genotype, BMI, and EF were significantly correlated with LVMI (Tables 4, 5). After adjusting by multivariate logistic regression, M235T remained statistically associated with LVMI, and TT-carrying subjects presented an average 8.6 (g/m^2^) higher LVMI than MT-carrying subjects (Table 6).

**Table 4:** The association between categorical variables and LVMI.

**Variables**	**LVMI (mean ± SD)**	***P-*value**
Gender		0.30
Male	90.4 ± 20.2	
Female	88.8 ± 21.9	
Use of antihypertensive medication		0.19
Yes	90.2 ± 21.2	
No	86.8 ± 18.9	
Use of ACE inhibitor		0.30
Yes	87.7 ± 17.6	
No	89.9 ± 21.2	
Use of angiotensin II receptor blockers		0.21
Yes	88.3 ± 19.9	
No	91.3 ± 21.5	
Use of calcium channel blockers		0.22
Yes	90.7 ± 19.9	
No	87.8 ± 21.4	
Use of diuretic		0.15
Yes	85.2 ± 19.3	
No	90.3 ± 20.7	
Use of beta-blockers		0.20
Yes	92.4 ± 21.6	
No	88.7 ± 20.3	
High salt intake		0.051
Yes	91.5 ± 21.4	
No	86.4 ± 19.5	
Alcohol use		0.09
Yes	91.7 ± 21.1	
No	87.6 ± 20.5	
Smoking		0.39
Yes	88.6 ± 22.5	
No	89.8 ± 20.6	
Sedentary lifestyle		0.32
Yes	88.7 ± 20.4	
No	90.2 ± 21.2	

*ACE, angiotensin-converting enzyme*.

**Table 5:** Univariate analysis between continuous variables and LVMI.

**Variables**	**R for correlation with LVMI**	***P-*value**
Genotype	0.152	0.037^*^
BMI	0.180	0.014^*^
Fasting plasma glucose	0.045	0.538
eGFR	0.096	0.193
Total cholesterol	0.086	0.857
HDL cholesterol	0.059	0.421
LDL cholesterol	0.002	0.980
Triglyceride	0.033	0.653
EF	0.248	0.001^*^
Duration of hypertension	0.229	0.002^*^

* *statistically significant*.

**Table 6:** Multivariate logistic regression analysis of LVMI.

**Variables**	**LVMI**	***P-*value**
TT vs. MT (g/m^2^)	8.6 ± 3.9	0.027^*^
Duration of hypertension (years)	1.1 ± 0.3	0.001^*^
BMI (kg/m^2^)	1.1 ± 0.5	0.041^*^
EF (%)	−0.9 ± 0.2	<0.001^*^

* *statistically significant*.

## Discussion

Echocardiography is a reliable, cost-effective imaging technique to determine LVM. Its adoption has been extensively certified in clinical practice, as well as research (37, 38). In this study, *AGT* M235T, duration of hypertension, BMI, and EF were found to be associated with calculated LVMI in Vietnamese patients diagnosed with essential hypertension. Factors that were considered to affect LVM, including age, gender, duration of hypertension, use of ACE inhibitors or angiotensin II receptor blockers, BMI, body surface area (BSA), and eGFR were not statistically different between TT and MT carriers. LVMI was significantly higher in TT compared to MT-carrying subjects even after adjusting for confounding factors, such as duration of hypertension, EF, and BMI. A similar association between M235T and LVM has been observed in Han Chinese (25) but not Russian and American populations (26, 27). The allele distribution of *AGT* M235T in Vietnamese hypertensive patients is similar to Nigerian patients, in whom the frequency of the M allele is <10% (17). The frequency of the M allele is 16% in Chinese, 19% in Japanese, 44% in Rumanian, and up to 67% in German hypertensive populations (18, 22, 24, 39). The predominance of the T allele and the profound association between the TT genotype and adverse cardiovascular phenotypes seem to be signature characteristics of Asian populations whose similarity in genetic landscapes has been reported (40–42). The TT genotype is also associated with a higher prevalence of hypertension in Malaysian and Japanese populations, but not Caucasians (18, 19, 24). Interestingly, a recent meta-analysis showed that the T allele is associated with cardiovascular diseases, including essential hypertension, myocardial infarction, and coronary artery disease in East Asians but not Caucasians (43). In addition to the effect on cardiomyocytes described above, greater level of Angiotensin II in TT carriers also affects endothelial cell function by multiple pathways, including promoting endothelial cell apoptosis, increasing vascular endothelial growth factor, and compromising nitric oxide production (44). These biological changes, in part, could explain why the T allele of M235T is associated with higher risk of essential hypertension, coronary artery disease, and heart failure, as well as mortality due to heart failure (45–47). However, these associations were inconsistent between ethnicities, and the mechanism underlying how M235T affects such cardiovascular diseases remains poorly understood (48). Remarkably, M235T does not directly regulate the transcription of *AGT* because it was proven not a hyperfunctional variant by expression study (49). The main driven variant controlling *AGT* transcription is A(−6)G. This variant, which is in strong linkage with M235T, is located in the promoter region of AGT and has been shown to be the key player in regulating *AGT* transcription (49). The lack of linkage disequilibrium between M235T and A(−6)G, therefore, may explain the inconsistent association between M235T and essential hypertension, coronary artery disease among certain ethnicities.

It has been proposed that the major T allele is associated with the upregulation of the angiotensinogen level and promotes sodium reabsorption, while the minor M allele is a recent “thrifty” mutation in humans to adapt to environmental changes in which sodium becomes widely available (50). The gradual increase in the frequency of the M allele in African, Southeast Asian, East Asian, and European populations may support the hypothesis that human ancestors originating from Africa migrated to Asia and then Europe (51). During the migration, the evolving “thrifty” M allele might have appeared and expanded. Together with the expansion of the M allele, the unfavorable cardiovascular phenotypes of the T allele have been fading.

This study has several limitations that need to be discussed. First, the relatively small sample size may lead to a bias in identifying the *AGT* M235T genotype in Vietnamese hypertensive subjects. Specifically, the MM genotype was not detectable in our data. Second, the measurement of LV by 2D ultrasound is less correlated with cardiac magnetic resonance in assessing LVM compared to real-time 3D echography (52). Third, like other polygenic models, the causative genetic factors affecting LVM are complex; *AGT* M235T *per se* cannot fully explain the difference in LVM between the genotype groups observed in this study. Therefore, further studies with larger sample sizes, comprehensive genetic analysis such as genome-wide association or whole genome sequencing, and advanced imaging techniques are required to better describe the association between genetic components and LVM in Vietnamese patients with essential hypertension.

To the best of our knowledge, this is the first study to determine the association between genetic variants and LVM in Vietnamese patients diagnosed with essential hypertension. The association between *AGT* M235T and LVM in Vietnamese hypertensive patients emphasizes once again the significant effect of this genetic polymorphism in left ventricular hypertrophy pathophysiology in Asian populations.

## Data Availability Statement

The original contributions presented in the study are included in the article/Supplementary Material, further inquiries can be directed to the corresponding author/s.

## Ethics Statement

The studies involving human participants were reviewed and approved by Ethics Committee of the University of Medicine and Pharmacy at Ho Chi Minh City, Vietnam. The patients/participants provided their written informed consent to participate in this study.

## Author Contributions

MD, TT, TM, TKT, and HC designed the study. TT, HT, and SH included the patients to the study. LL, HV, and TM performed genotyping procedure. MD, TT, HT, SH, TKT, and HC analyzed the data. MD, TT, and TM wrote the manuscript. All authors critically revised the manuscript.

## Conflict of Interest

The authors declare that the research was conducted in the absence of any commercial or financial relationships that could be construed as a potential conflict of interest.

## Supplementary Material

Supplementary Figure 1 Represented image of how echocardiography measurements were documented. IVSd, interventricular septal thickness at end-diastole; IVSs, interventricular septal thickness in systole; LPWd, posterior wall thickness at end-diastole; LPWs, posterior wall thickness in systole; LVIDd, left ventricular internal dimension at end-diastole; LVIDs, left ventricular internal dimension in systole.

Supplementary Table 1 Primers for AGT M235T genotyping.

## Abbreviations

ACE: angiotensin-converting enzyme
AGT: angiotensinogen
BMI: body mass index
BSA: body surface area
EF: ejection fraction
GFR: glomerular filtration rate
IVSd: interventricular septal thickness at end-diastole
IVSs: interventricular septal thickness in systole
LPWd: posterior wall thickness at end-diastole
LPWs: posterior wall thickness in systole
LVIDd: left ventricular internal dimension at end-diastole
LVIDs: left ventricular internal dimension in systole
LVM: left ventricular mass
LVMI: left ventricular index
RAA: renin-angiotensin-aldosterone
RWT: relative wall thickness.
