# Supplementary figures and images for: Association Between AGT M235T and Left Ventricular Mass in Vietnamese Patients Diagnosed With Essential Hypertension
Source: Front Cardiovasc Med. 2021 Feb 19;8:608948. doi: 10.3389/fcvm.2021.608948 (PMC7933009; doi:10.3389/fcvm.2021.608948)

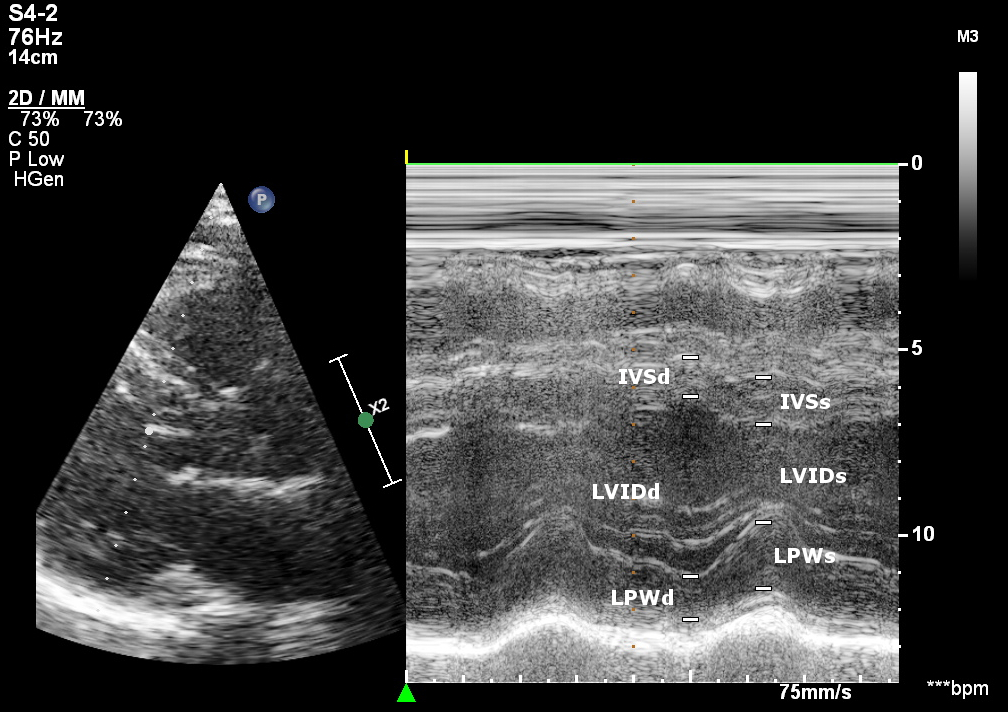

Supplement: Supplementary Figure 1 — Represented image of how echocardiography measurements were documented. IVSd, interventricular septal thickness at end-diastole; IVSs, interventricular septal thickness in systole; LPWd, posterior wall thickness at end-diastole; LPWs, posterior wall thickness in systole; LVIDd, left ventricular internal dimension at end-diastole; LVIDs, left ventricular internal dimension in systole. [file Image_1.JPEG]
