# Supplementary material for: Association Between AGT M235T and Left Ventricular Mass in Vietnamese Patients Diagnosed With Essential Hypertension
Source: Front Cardiovasc Med. 2021 Feb 19;8:608948. doi: 10.3389/fcvm.2021.608948 (PMC7933009; doi:10.3389/fcvm.2021.608948)
Supplement: Supplementary Table 1 — Primers for AGT M235T genotyping. [file Table_1.DOCX]

Supplementary Material

Supplementary Table 1. Primers for *AGT* M235T genotyping

| **Primers** | **Sequence (5’ to 3’)** | **Mix ratio** |
| --- | --- | --- |
| AGT-F | 5’-CCACGCTCTCTGGACTTCAC-3’ | 1 |
| AGT-R | 5’-TTCTCAAGGGTGGTCACCAG-3’ | 2 |
| AGT-WT-F | 5’-AAGACTGGCTGCTCCCTGAT-3’ | 2 |
| AGT-699C-R2 | 5’-TGCTGTCCACACTGGCTCTCA-3’ | 1 |
